# Supplementary material for: Fighting Microbial Infections from Escherichia coli O157:H7: The Combined Use of Three Essential Oils of the Cymbopogon Genus and a Derivative of Esculentin-1a Peptide
Source: Antibiotics (Basel). 2024 Jan 16;13(1):86. doi: 10.3390/antibiotics13010086 (PMC10812396; doi:10.3390/antibiotics13010086)
Supplement: Supplementary file 1 [file antibiotics-13-00086-s001.zip › antibiotics-2809509-supplementary.pdf]

**Table S1 Supplementary Materials**: Chemical compositions (%) of essential oils.

| **Component**[a] | **RI**[b] | **RI Lit.**[c] | ***C. citratus*** **(%) ± S.D.** | ***C. flexuosus*** **(%) ± S.D.** | ***C. martinii*** **(%) ± S.D.** |
|---|---|---|---|---|---|
| tricyclene | 921 | 921 | 0.15 ± 0.00 | 0.17 ± 0.00 | - |
| *α*-pinene | 932 | 932 | 0.24 ± 0.01 | 0.14 ± 0.01 | - |
| camphene | 946 | 952 | 1.94 ± 0.03 | 1.81 ± 0.01 | - |
| 6-methyl-5-hepten-2-one | 986 | 988 | 1.61 ± 0.00 | 1.01 ± 0.04 | - |
| limonene | 1028 | 1024 | 0.27 ± 0.01 | 0.84 ± 0.02 | tr[d] |
| (*Z)*-*β*-ocimene | 1038 | 1032 | 0.18 ± 0.01 | 0.19 ± 0.02 | 0.21 ± 0.03 |
| (*E*)-*β*-ocimene | 1048 | 1044 | tr | tr | 1.37 ± 0.06 |
| pentyl propyl ketone | 1073 | 1078 | 1.20 ± 0.01 | 0.82 ± 0.02 | - |
| terpinolene | 1087 | - | tr | - | - |
| linalool | 1099 | 1095 | 0.75 ± 0.05 | 0.66 ± 0.07 | 2.32 ± 0.15 |
| *exo*-isocitral | 1145 | 1145 | 0.65 ± 0.01 | 0.68 ± 0.07 | - |
| *trans*-chrysanthemal | 1149 | 1153 | - | 0.14 ± 0.01 | - |
| citronellal | 1154 | 1148 | 0.11 ± 0.01 | tr | - |
| borneol | 1164 | 1165 | 0.16 ± 0.01 | 0.19 ± 0.07 | - |
| (Z)-*iso*-citral | 1165 | 1165 | 1.35 ± 0.08 | 1.55 ± 0.10 | - |
| *iso*-geranial | 1183 | 1184 | 2.10 ± 0.10 | 2.52 ± 0.24 | - |
| *α*-terpineol | 1190 | 1186 | tr | tr | - |
| neral | 1241 | 1235 | 33.84 ± 0.02 | 33.22 ± 0.05 | - |
| geraniol | 1255 | 1249 | 4.63 ± 0.10 | 5.17 ± 0.21 | 81.41 ± 0.57 |
| geranial | 1271 | 1267 | 42.83 ± 0.20 | 42.43 ± 0.30 | 0.39 ± 0.00 |
| geranyl acetate | 1385 | 1379 | 3.49 ± 0.08 | 3.29 ± 0.12 | 11.74 ± 0.18 |
| (*E*)-caryophyllene | 1420 | 1417 | 1.83 ± 0.02 | 2.78 ± 0.02 | 2.11 ± 0.03 |
| *γ*-cadinene | 1515 | 1513 | 1.52 ± 0.05 | 1.42 ± 0.05 | - |
| *δ*-cadinene | 1525 | 1522 | 0.19 ± 0.01 | 0.20 ± 0.02 | - |
| Total identified (%) | | | 99.27 | 99.43 | 99.59 |
| Grouped components (%) | | | | | |
| Monoterpene hydrocarbons | | | 2.94 | 3.23 | 1.62 |
| Oxygenated monoterpenes | | | 89.98 | 89.97 | 95.86 |
| Sesquiterpene hydrocarbons | | | 2.81 | 1.83 | 2.11 |
| Others | | | 3.54 | 4.4 | - |

a) Elution order according to t**he** HP-5MS column (30 m l. x 0.25 mm i.d., 0.1 mm f.t.); b) temperature-programmed linear retention index calculated using a $C_8$-$C_{30}$ mixture of alkanes; c) retention index taken from the ADAMS library; d) traces, % < 0.1.
